# Prevalence of diabetes and prediabetes among working-age adults and influencing factors of new-onset diabetes: a five-year cohort study (2018–2023)

**DOI:** 10.3389/fendo.2025.1626925

**Published:** 2025-09-17

**Authors:** Jing Tan, Mingzhu Chen, Yang Lei, Xiaofen Shi, Cuiping Cao, Naili Du, Yuyou Yao, Xiaojuan Yao

**Affiliations:** ^1^ Department of Endocrine, Wuxi People’s Hospital, The Affiliated Wuxi People’s Hospital of Nanjing Medical University and Wuxi Medical Center, Nanjing Medical University, Wuxi, China; ^2^ Department of Clinical Nursing, School of Nursing Nanjing Medical University, Nanjing, China; ^3^ Department of Internal Medicine Ward, Wuxi People’s Hospital, The Affiliated Wuxi People’s Hospital of Nanjing Medical University and Wuxi Medical Center Nanjing Medical University, Wuxi, China; ^4^ Department of Health Management Center, Wuxi People’s Hospital, The Affiliated Wuxi People’s Hospital of Nanjing Medical University and Wuxi Medical Center, Nanjing Medical University, Wuxi, China; ^5^ Department of Emergency Medicine, Wuxi People’s Hospital, The Affiliated Wuxi People’s Hospital of Nanjing Medical University and Wuxi Medical Center, Nanjing Medical University, Wuxi, China

**Keywords:** diabetes, prediabetes, predictive model, nomogram, generalized estimating equations

## Abstract

**Background:**

Diabetes and prediabetes in the young and middle-aged population represent a significant public health challenge in China. In recent years, the prevalence of diabetes has gradually increased within this group. This study aims to evaluate the prevalence of diabetes and prediabetes in a health check-up population of young and middle-aged individuals, and to analyze the key factors influencing the new onset of diabetes. The study provides data support for the early prevention of diabetes.

**Method:**

This study used retrospective cohort analyses to examine the data from the physical examination centers of three hospitals in Wuxi, China, for the population aged 18–59 from 2018 to 2023. Analyzing the changes in the prevalence of diabetes and prediabetes in the population. Single-factor analysis was used to examine differences in basic characteristics and laboratory indicators between individuals who developed diabetes and those who did not within five years. A multifactorial logistic regression model (MLR model), Cox proportional hazards model (Cox model), and generalized estimating equation (GEE) model were employed to analyze the factors associated with the development of diabetes. ROC curves were used to evaluate the performance of these three models. Finally, a nomogram was constructed to predict the risk of developing diabetes in the next five years.

**Results:**

From 2018 to 2023, the number of diabetes cases increased year by year, with the highest increase of 1.39% observed between 2020 and 2021. New-onset diabetes patients had poorer lifestyle and health profiles compared to those without new-onset diabetes. New-onset diabetes group also had worse metabolic and inflammatory profiles (P < 0.05), with significantly lower eGFR (P = 0.027). The AUC values for all three models were 0.64, with the GEE model performing best in Youden index (0.237), the Cox model in sensitivity (0.577), and the MLR model in specificity (0.776). The most significant factors identified were NLR, FBG, Cr, BMI, and exercise habits. The nomogram built using these five factors showed good predictive performance with AUC values of 0.705 and 0.666 in the training and test sets, respectively.

**Conclusion:**

The significant factors influencing the onset of diabetes include NLR, FPG, Cr, BMI, and exercise habits. The nomogram can effectively predict the risk of diabetes in the next five years, providing a powerful tool for early intervention. Future research could explore the interactions among these factors and validate the model’s applicability in different populations.

## 1 Introduction

Diabetes Mellitus (DM) is a chronic disease with a growing prevalence, posing a significant health burden, especially among young and middle-aged individuals (1, 2). DM is associated with high disability and mortality rates, and, despite advancements in treatment, there is currently no cure (3). In 2021, approximately 536.6 million people aged 20–79 worldwide had diabetes, and this number is projected to reach 783.2 million by 2045, with higher prevalence in urban and high-income regions (4). With improvements in living standards, dietary patterns in the country have shifted to high sugar, high fat, and high-energy foods, while the widespread use of mechanization and automation has led to reduced physical activity and exercise, contributing to the annual rise in the incidence and number of diabetes patients in China (5).

Middle-aged and young patients have become an important and often overlooked group among those with type 2 diabetes mellitus (T2DM) (6, 7). As the backbone of society and family, they face high life and work pressures, heavy psychological burdens, and often neglect their own health. With relatively low self-management abilities and insufficient role management, they are at a higher risk of accelerated complications and worsened psychological disorders. Therefore, early screening of individuals at risk for prediabetes and the implementation of targeted preventive measures during the early stages of the disease are crucial for reducing both the incidence of diabetes and its associated societal burden.

Prediabetes (PDM) is a condition in which blood glucose levels are higher than normal but below the diagnostic threshold for diabetes (8, 9). In China, the prevalence of prediabetes among adults ranges from 35.7% to 50.1%. The prediabetes population serves as the “reserve force” for diabetes, with the risk of progression to diabetes being 2.7 times higher than that of the general population. It is estimated that 5% to 10% of prediabetic individuals will develop type 2 diabetes each year (10, 11). However, this stage is reversible, and early intervention can effectively delay or even prevent the onset of diabetes. Therefore, the development and validation of risk prediction models is crucial. By identifying high-risk populations early, these models can provide the basis for precise interventions and personalized management, helping to reduce the incidence of diabetes and its related complications. Additionally, predictive models help optimize the allocation of public health resources, prioritize high-risk groups, enhance diagnostic and treatment efficiency, reduce the burden on the healthcare system, and ultimately improve overall health outcomes.

A comprehensive assessment of diabetes onset risk involves evaluating biomarkers related to glucose metabolism, lipid metabolism, inflammation, and organ function. Fasting plasma glucose (FPG), glycated hemoglobin (HbA1c), and fasting insulin (FI) directly reflect blood glucose control. Total cholesterol (TC) and low-density lipoprotein (LDL) reflect lipid metabolism, with lipid imbalance being an important risk factor for diabetes (12, 13). Hypertension is closely related to diabetes and insulin resistance, making blood pressure a key indicator for diabetes prediction (14). Elevated total bilirubin is associated with insulin resistance and diabetes, possibly reflecting liver damage or oxidative stress (15). Declining kidney function significantly increases the risk of diabetes, and estimated glomerular filtration rate (eGFR) is an important indicator for assessing kidney function (16). Finally, white blood cell count (WBC), neutrophil-to-lymphocyte ratio (NLR), and platelet-to-lymphocyte ratio (PLR) reflect the inflammatory status, with chronic low-grade inflammation being a significant pathological mechanism of diabetes (17, 18).

At present, there have been many studies on the risk factors for the onset or prevalence of diabetes (19, 20), but most of the results are based on cross-sectional surveys, which have weak causal demonstration strength (21, 22). A small number of cohort studies, with small sample sizes, have not fully considered the annual changes in the prevalence of diabetes. To address this limitation, we explored the factors associated with the onset of new diabetes within 1, 3, and 5 years through the generalized estimating equations (GEE) model. This dynamic study approach enhanced the accuracy and generalizability of the results. The purpose of this study is to analyze the data from the physical examination centers of three hospitals in Wuxi, China, from 2018 to 2023, explore the changes in the prevalence of diabetes and prediabetes among young and middle-aged individuals, build prediction models for diabetes incidence, and evaluate the prediction performance of different models.

## 2 Materials and methods

### 2.1 Study design and data sources

The study population comes from the health examination databases of three hospitals in Wuxi, China, covering individuals aged 18–59 years from 2018 to 2023. The database used in this study is a relational database based on Structured Query Language (SQL). The data includes basic information such as: outpatient number (unique code), gender, age, region, marital status, occupation, etc. Disease diagnosis: the prevalence of major diseases in the examined individuals and their corresponding International Classification of Diseases (ICD), and whether the disease was newly diagnosed in the current year’s health examination. Disease treatment: whether the major disease in the examined individuals had been treated. Physical examination and laboratory indicators: physical examination includes height, weight, heart rate, systolic pressure, and diastolic pressure. Laboratory indicators include blood glucose, lipid profile, liver function, kidney function, and others. Inclusion criteria: 1) Aged 18 to 59 years; 2) Underwent physical examinations in three hospitals between 2018 and 2023 and provided complete clinical and laboratory data. Exclusion criteria: 1) Severe systemic diseases such as advanced cancer or major immune deficiency disorders; 2) Severe liver or kidney dysfunction; 3) Severe psychiatric disorders or poor compliance; 4) Missing key data, such as diabetes status.

According to the standards of the World Health Organization (WHO) and the American Diabetes Association (ADA), diabetes is diagnosed if the random venous plasma glucose concentration is ≥11.1 mmol/L, fasting blood glucose concentration is ≥7.0 mmol/L, or the 2-hour post-glucose load blood glucose concentration is ≥11.1 mmol/L. Patients who were not diagnosed with diabetes at the start of the study but were first diagnosed with diabetes during the follow-up period are defined as having “new-onset diabetes”.

### 2.2 Data collection and laboratory standardization

All participants were screened and data was collected by trained professionals following a unified protocol. The information was stored and managed through a centralized electronic health record system. The laboratories in all three hospitals used the same testing equipment and methods, and employed standardized reagents and procedures. To ensure consistency and reliability of the laboratory data, regular equipment calibration and quality control were conducted. Additionally, all three hospitals adhered to the same diagnostic criteria for diabetes.

### 2.3 Prevalence analysis

Evaluating the changes in the prevalence of diabetes and prediabetes in different years from 2018 to 2023. The prevalence rate was calculated by dividing the number of newly diagnosed diabetes cases and individuals already diagnosed with diabetes by the total number of individuals.

### 2.4 Retrospective cohort analysis

In analyzing newly developed diabetes, individuals who already had diabetes in 2018 were excluded. Differences in baseline characteristics and laboratory indicators between individuals with newly developed diabetes and those without within 5 years were compared. For continuous variables, normality testing is conducted prior to univariate analysis. If the data follow a normal distribution, a t-test is used; otherwise, the Mann-Whitney U test is applied. For categorical variables, the chi-square test is used. We conducted multicollinearity analysis on the explanatory variables in the models by calculating the variance inflation factor (VIF) for each variable. The dataset was split into a training set and a testing set in a 7:3 ratio. In the training set, multivariate logistic regression (MLR model), Cox proportional hazard models (Cox model), and generalized estimation equation models (GEE model) were used for further analysis to assess the independent effects of various factors on the new onset of diabetes. Multivariate Logistic Regression (MLR): This model is suitable for analyzing binary outcome variables and can effectively assess the independent effects of various factors on the onset of new diabetes, providing the corresponding odds ratios (ORs). It is the primary model used in this study for analyzing new-onset diabetes. Cox Proportional Hazards Model: Considering that the onset of diabetes has a time-dependent characteristic, the Cox model is used for survival analysis to explore risk factors for new-onset diabetes over different time periods. It accounts for the timing of the event occurrence and potential time effects. Generalized Estimating Equation (GEE): GEE is particularly suited for handling repeated measures data or correlated observations. In this study, data on diabetes diagnoses at multiple time points (1 year, 3 years, and 5 years) for the same patients were collected. Therefore, the GEE model can effectively account for the correlation within these data and provide robust estimates. By integrating these three models, we can comprehensively evaluate the factors influencing the onset of new diabetes from multiple perspectives, ensuring the applicability of the results. Receiver operating characteristic (ROC) curve analysis was used to evaluate the predictive performance of the multivariate models, and the performance of the models was compared in terms of sensitivity, specificity, and Youden index. A nomogram for predicting the risk of diabetes in the next five years was constructed based on significant influencing factors in the training set and validated in the testing set. All analyses were conducted using R 4.4.0. The research flowchart is shown in **Supplementary Figure 1**.

## 3 Results

### 3.1 Trend of diabetes and prediabetes prevalence among young and middle-aged health checkup population from 2018 to 2023

**Figure 1:**
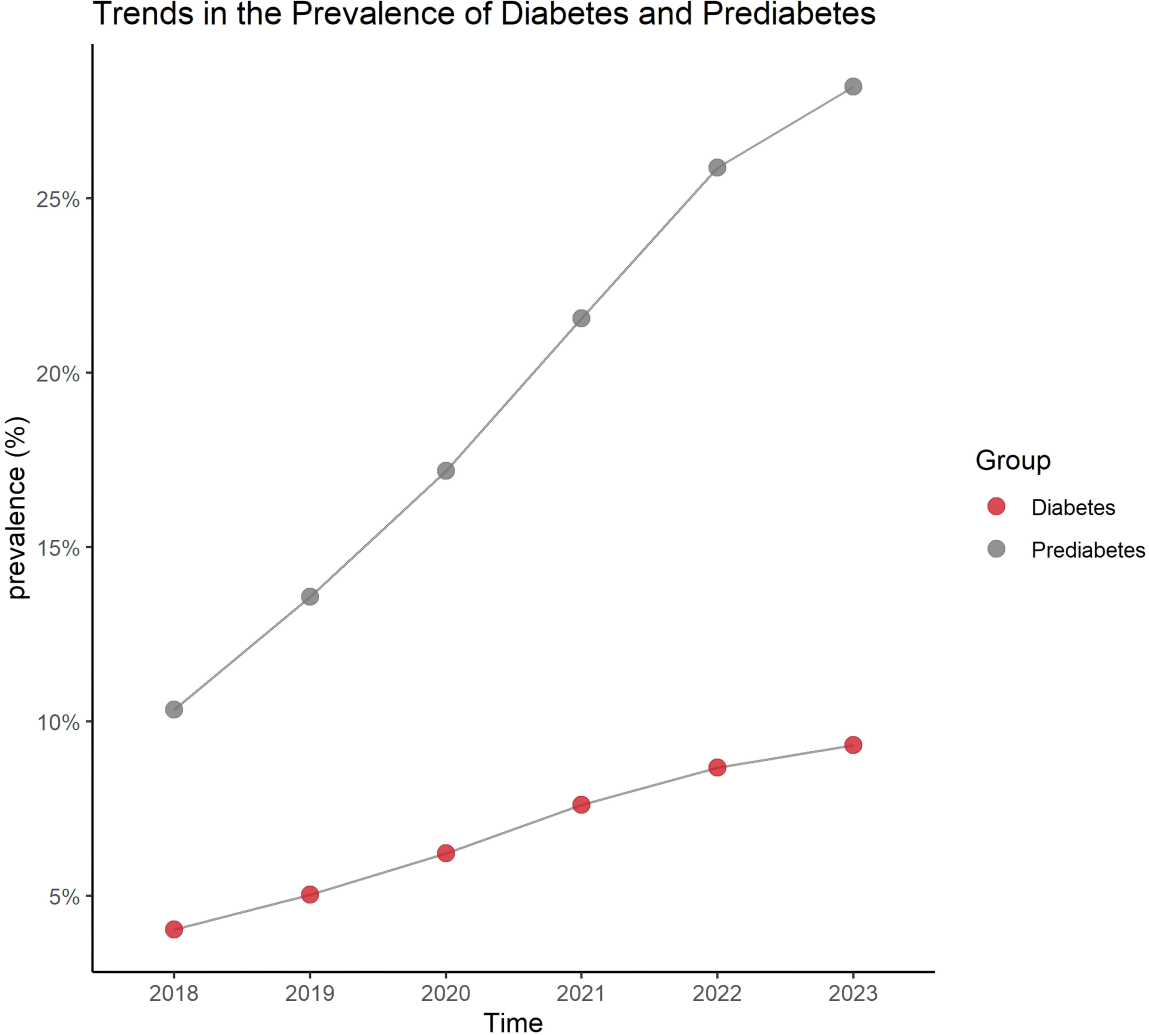
Trend of diabetes and prediabetes prevalence from 2018 to 2023.

The results show that from 2018 to 2023, the number of individuals diagnosed with diabetes and prediabetes has shown a gradual increase year by year, with the largest rise occurring between 2020 and 2021 (**Figure 1**).

### 3.2 Baseline characteristic differences between newly diagnosed diabetes patients and non-newly diagnosed diabetes patients over 5 years

A total of 450 new cases were identified within the 5-year period. The age range of the entire population was 18–59 years. Male patients accounted for 52.08%, and female patients accounted for 47.92%. The BMI was 26.6 (17.3-35.9), with the BMI of newly diagnosed diabetes patients being 26.7 (17.3-35.9) and that of non-newly diagnosed patients being 25.8 (17.4-35.9). The p-value was 0.0415, indicating that the BMI of newly diagnosed diabetes patients was significantly higher than that of non-newly diagnosed patients. Among the patients, 40.41% were manual laborers, 37.85% were intellectual laborers, and 21.73% were service workers. Patients with a family history of diabetes accounted for 21.67%. Those with a history of cardiovascular disease accounted for 3.3%, while those with a history of kidney disease accounted for 0.78%. Low-income patients accounted for 46.92%, moderate-income patients accounted for 41.17%, and high-income patients accounted for 11.9%. Urban residents accounted for 77.57%, while rural residents accounted for 22.43%. The p-value was 0.0064, indicating that 82.89% of newly diagnosed diabetes patients were urban residents, which was significantly higher than the 77.26% of non-newly diagnosed patients. Mild smokers accounted for 68.29%, moderate smokers accounted for 29.5%, and heavy smokers accounted for 2.21%. Mild drinkers accounted for 67.72%, moderate drinkers accounted for 30.62%, and heavy drinkers accounted for 1.65%. 48.06% of patients exercised regularly, 30.24% exercised occasionally, and 21.7% were inactive. The p-value was 0.0138, indicating that a higher proportion of newly diagnosed diabetes patients (25.56%) were inactive compared to non-newly diagnosed patients (**Table 1**).

**Table 1:** Baseline characteristics differences between newly diagnosed diabetes patients and non-newly diagnosed patients within 5 years.

	All Patient (n=8158)	5-year incidence (n=450)	5-year non-incidence (n=7708)	P-value
Age	38 (18-59)	38 (18-59)	38 (18-59)	0.455
Gender				0.2112607
Male	4249 (52.08%)	221 (49.11%)	4028 (52.26%)	
Female	3909 (47.92%)	229 (50.89%)	3680 (47.74%)	
BMI	26.6 (17.3-35.9)	26.7 (17.3-35.9)	25.8 (17.4-35.9)	0.0415
Occupation Type				0.3794144
Manual Labor	3297 (40.41%)	189 (42%)	3108 (40.32%)	
Intellectual Labor	3088 (37.85%)	175 (38.89%)	2913 (37.79%)	
Service Labor	1773 (21.73%)	86 (19.11%)	1687 (21.89%)	
Family History of Diabetes				0.4115642
Yes	1768 (21.67%)	105 (23.33%)	1663 (21.57%)	
No	6390 (78.33%)	345 (76.67%)	6045 (78.43%)	
History of Cardiovascular Disease				0.1241267
Yes	269 (3.3%)	21 (4.67%)	248 (3.22%)	
No	7889 (96.7%)	429 (95.33%)	7460 (96.78%)	
History of Kidney Disease				0.5939934
Yes	64 (0.78%)	5 (1.11%)	59 (0.77%)	
No	8094 (99.22%)	445 (98.89%)	7649 (99.23%)	
Income Level				0.1087283
Low	3828 (46.92%)	227 (50.44%)	3601 (46.72%)	
Middle	3359 (41.17%)	182 (40.44%)	3177 (41.22%)	
High	971 (11.9%)	41 (9.11%)	930 (12.07%)	
Residential Area				0.00641766
Urban	6328 (77.57%)	373 (82.89%)	5955 (77.26%)	
Rural	1830 (22.43%)	77 (17.11%)	1753 (22.74%)	
Smoking				0.6188209
Mild	5571 (68.29%)	298 (66.22%)	5273 (68.41%)	
Moderate	2407 (29.5%)	141 (31.33%)	2266 (29.4%)	
Severe	180 (2.21%)	11 (2.44%)	169 (2.19%)	
Drinking				0.2386928
Mild	5525 (67.72%)	307 (68.22%)	5218 (67.7%)	
Moderate	2498 (30.62%)	140 (31.11%)	2358 (30.59%)	
Severe	135 (1.65%)	3 (0.67%)	132 (1.71%)	
Exercise Habits				0.01376978
Regular Exercise	3921 (48.06%)	187 (41.56%)	3734 (48.44%)	
Occasional Exercise	2467 (30.24%)	148 (32.89%)	2319 (30.09%)	
Lack of Exercise	1770 (21.7%)	115 (25.56%)	1655 (21.47%)	

### 3.3 Laboratory indicator differences between newly diagnosed diabetes patients and non-newly diagnosed diabetes patients over 5 years

There were no significant differences in waist circumference, systolic blood pressure, diastolic blood pressure, low-density lipoprotein, or high-density lipoprotein between the two groups. The newly diagnosed diabetes group showed significantly higher levels of fasting blood glucose, glycated hemoglobin, total cholesterol, triglycerides, fasting insulin, total bilirubin, serum creatinine, white blood cell count, neutrophil/lymphocyte ratio, and platelet/lymphocyte ratio compared to the non-newly diagnosed group (P < 0.05). In contrast, the estimated glomerular filtration rate was significantly lower in the newly diagnosed group (P < 0.05) (**Table 2**).

**Table 2:** Laboratory indicator differences between newly diagnosed diabetes patients and non-newly diagnosed patients within 5 years.

	All Patient (n=8158)	5-year incidence (n=450)	5-year non-incidence (n=7708)	P-value
Waist Circumference (cm)	89.1 (65.0-113.0)	88.9 (65.1-112.7)	89.1 (65.0-113.0)	0.695
Systolic Blood Pressure, SBP (mmHg)	117.4 (102.0-133.0)	116.8 (102.2-132.9)	117.4 (102.0-133.0)	0.652
Diastolic Blood Pressure, DBP (mmHg)	77.4 (57.0-98.0)	76.7 (57.0-98.0)	77.5 (57.0-98.0)	0.307
Fasting Blood Glucose, FBG (mmol/L)	4.9 (3.1-6.7)	5.1 (3.1-6.7)	4.9 (3.1-6.7)	0.00425
Glycated Hemoglobin, HbA1c (%)	5.2 (4.1-6.3)	5.3 (4.1-6.3)	5.2 (4.1-6.3)	0.00288
Total Cholesterol, TC (mmol/L)	4.4 (2.5-6.2)	4.6 (2.5-6.2)	4.3 (2.5-6.2)	0.0105
Low-Density Lipoprotein, LDL (mmol/L)	2.3 (1.2-3.5)	2.4 (1.2-3.5)	2.3 (1.2-3.5)	0.32
High-Density Lipoprotein, HDL (mmol/L)	1.8 (0.7-2.9)	1.8 (0.7-2.9)	1.8 (0.7-2.9)	0.129
Triglycerides, TG (mmol/L)	1.5 (0.8-2.3)	1.6 (0.8-2.3)	1.5 (0.8-2.3)	0.049
Fasting Insulin, FI (µU/mL)	14.9 (2.8-27.3)	16.0 (2.9-27.3)	14.9 (2.8-27.3)	0.0448
Total Bilirubin, TBIL (μmol/L)	11.4 (3.6-18.8)	12.1 (3.6-18.7)	11.3 (3.6-18.8)	0.0404
Serum Creatinine, Cr (μmol/L)	79.0 (46.2-112.8)	83.4 (46.2-112.6)	78.8 (46.2-112.8)	0.00444
Estimated Glomerular Filtration Rate, eGFR (mL/min/1.73m²)	100.9 (83.4-118.8)	98.1 (83.6-118.7)	101.0 (83.4-118.8)	6.93E-05
White Blood Cell, WBC (×10^9/L)	7.7 (4.4-10.8)	7.9 (4.4-10.8)	7.6 (4.4-10.8)	0.00439
Neutrophil-to-Lymphocyte Ratio, NLR	2.3 (1.2-3.4)	2.5 (1.2-3.4)	2.3 (1.2-3.4)	0.00175
Platelet-to-Lymphocyte Ratio, PLR	210.0 (105.4-314.3)	219.8 (105.5-314.3)	209.6 (105.4-314.2)	0.00165

### 3.4 Multivariable logistic regression model, Cox proportional hazards model, and generalized estimating equations to evaluate factors influencing new-onset diabetes within 5 years

The VIF for all explanatory variables was less than 5 (**Supplementary Table 1**), indicating that there is no significant multicollinearity problem between the variables. Therefore, we can conclude that the explanatory variables in our models are not subject to multicollinearity, ensuring the stability of the estimation results. In the training set, we conducted further multivariable analysis of the significant factors identified in Sections 3.1 and 3.2. The results showed that in the MLR model, body mass index (BMI, OR=1.016, 95% CI: 1.005-1.027, p=0.004), fasting plasma glucose (FPG, OR=1.009, 95% CI: 1.003-1.014, p=0.003), fasting insulin (FI, OR=1.001, 95% CI: 1.000-1.002, p=0.024), creatinine (Cr, OR=1.000, 95% CI: 1.000-1.001, p=0.006), white blood cell count (WBC, OR=1.004, 95% CI: 1.000-1.007, p=0.026), neutrophil-to-lymphocyte ratio (NLR, OR=1.015, 95% CI: 1.006-1.025, p=0.001), and residential area (OR=1.018, 95% CI: 1.004-1.032, p=0.014) were significantly positively associated with the risk of new-onset diabetes. The higher the levels of these factors, the higher the risk of diabetes. Estimated glomerular filtration rate (eGFR, OR=0.999, 95% CI: 0.999-1.000, p=0.027) and exercise habits (OR=0.992, 95% CI: 0.985-0.999, p=0.028) were significantly negatively associated with the occurrence of diabetes, suggesting that higher eGFR and regular exercise may reduce the risk of new-onset diabetes. In the Cox model, BMI (HR=1.162, 95% CI: 1.052-1.284, p=0.003), FPG (HR=1.179, 95% CI: 1.058-1.313, p=0.003), FI (HR=1.019, 95% CI: 1.003-1.035, p=0.022), Cr (HR=1.008, 95% CI: 1.003-1.014, p=0.005), WBC (HR=1.071, 95% CI: 1.008-1.138, p=0.028), NLR (HR=1.339, 95% CI: 1.120-1.602, p=0.001), and residential area (HR=1.459, 95% CI: 1.080-1.972, p=0.014) were significant positive risk factors for new-onset diabetes, indicating that these factors may increase the risk of developing diabetes. eGFR (HR=0.988, 95% CI: 0.977-0.999, p=0.029) and exercise habits (HR=0.854, 95% CI: 0.744-0.981, p=0.025) were significant negative risk factors for new-onset diabetes, suggesting that higher eGFR and regular exercise may reduce the risk. In the GEE model, BMI (estimate = 0.019, P < 0.001), FPG (estimate = 0.158, P < 0.001), HbA1c (estimate = 0.221, P < 0.001), TC (estimate = 0.120, P < 0.001), FI (estimate = 0.012, P = 0.017), TBIL (estimate = 0.022, P = 0.007), Cr (estimate = 0.008, P < 0.001), WBC (estimate = 0.061, P < 0.001), NLR (estimate = 0.233, P < 0.001), and PLR (estimate = 0.002, P < 0.001) were all positively correlated with the risk of new-onset diabetes. eGFR (estimate = -0.017, P < 0.001) and exercise habits (estimate = -0.195, P < 0.001) were negatively correlated with the risk of new-onset diabetes (**Table 3**).

**Table 3:** Logistic regression, cox proportional hazards model, and generalized estimating equations to assess factors influencing the 5-year incidence of newly diagnosed diabetes.

MLR Model							
term	estimate	std error	statistic	p value	OR	CI-lower	CI-upper
BMI	0.016	0.006	2.863	0.004	1.016	1.005	1.027
FBG	0.009	0.003	3.017	0.003	1.009	1.003	1.014
HbA1c	0.008	0.005	1.610	0.107	1.008	0.998	1.017
TC	0.005	0.003	1.625	0.104	1.005	0.999	1.010
TG	0.013	0.007	1.936	0.053	1.014	1.000	1.027
FI	0.001	0.000	2.258	0.024	1.001	1.000	1.002
TBIL	0.001	0.001	1.396	0.163	1.001	1.000	1.002
Cr	0.000	0.000	2.767	0.006	1.000	1.000	1.001
eGFR	-0.001	0.000	-2.218	0.027	0.999	0.999	1.000
WBC	0.004	0.002	2.234	0.026	1.004	1.000	1.007
NLR	0.015	0.005	3.209	0.001	1.015	1.006	1.025
PLR	0.000	0.000	1.867	0.062	1.000	1.000	1.000
Residential Area	0.018	0.007	2.468	0.014	1.018	1.004	1.032
Exercise Habits	-0.008	0.004	-2.198	0.028	0.992	0.985	0.999

Cox Model							
term	estimate	std error	statistic	p value	OR	CI-lower	CI-upper
BMI	0.150	0.051	2.957	0.003	1.162	1.052	1.284
FBG	0.165	0.055	2.992	0.003	1.179	1.058	1.313
HbA1c	0.147	0.091	1.614	0.106	1.158	0.969	1.383
TC	0.089	0.054	1.659	0.097	1.093	0.984	1.214
TG	0.252	0.134	1.882	0.060	1.286	0.990	1.671
FI	0.019	0.008	2.295	0.022	1.019	1.003	1.035
TBIL	0.018	0.013	1.369	0.171	1.018	0.992	1.044
Cr	0.008	0.003	2.820	0.005	1.008	1.003	1.014
eGFR	-0.012	0.006	-2.190	0.029	0.988	0.977	0.999
WBC	0.068	0.031	2.201	0.028	1.071	1.008	1.138
NLR	0.292	0.091	3.197	0.001	1.339	1.120	1.602
PLR	0.002	0.001	1.789	0.074	1.002	1.000	1.004
Residential Area	0.378	0.154	2.458	0.014	1.459	1.080	1.972
Exercise Habits	-0.158	0.071	-2.234	0.025	0.854	0.744	0.981

GEE Model							
term	Estimate	Naive S E	Naive z	Robust S E	Robust z		
BMI	0.192	0.007	4.881	0.007	4.834		
FBG	0.158	0.034	4.625	0.035	4.474		
HbA1c	0.221	0.056	3.924	0.056	3.920		
TC	0.120	0.033	3.589	0.033	3.655		
TG	0.109	0.083	1.326	0.084	1.304		
FI	0.012	0.005	2.372	0.005	2.388		
TBIL	0.022	0.008	2.707	0.008	2.690		
Cr	0.008	0.002	4.046	0.002	4.007		
eGFR	-0.017	0.004	-4.709	0.003	-4.724		
WBC	0.061	0.019	3.162	0.019	3.279		
NLR	0.233	0.056	4.119	0.056	4.164		
PLR	0.002	0.001	3.692	0.001	3.760		
Residential Area	0.434	0.097	4.481	0.096	4.506		
Exercise Habits	-0.195	0.044	-4.472	0.043	-4.560		

|Z| > 1.96 indicates statistical significance (p < 0.05).

### 3.5 Model performance evaluation and selection of significant risk factors

**Figure 2:**
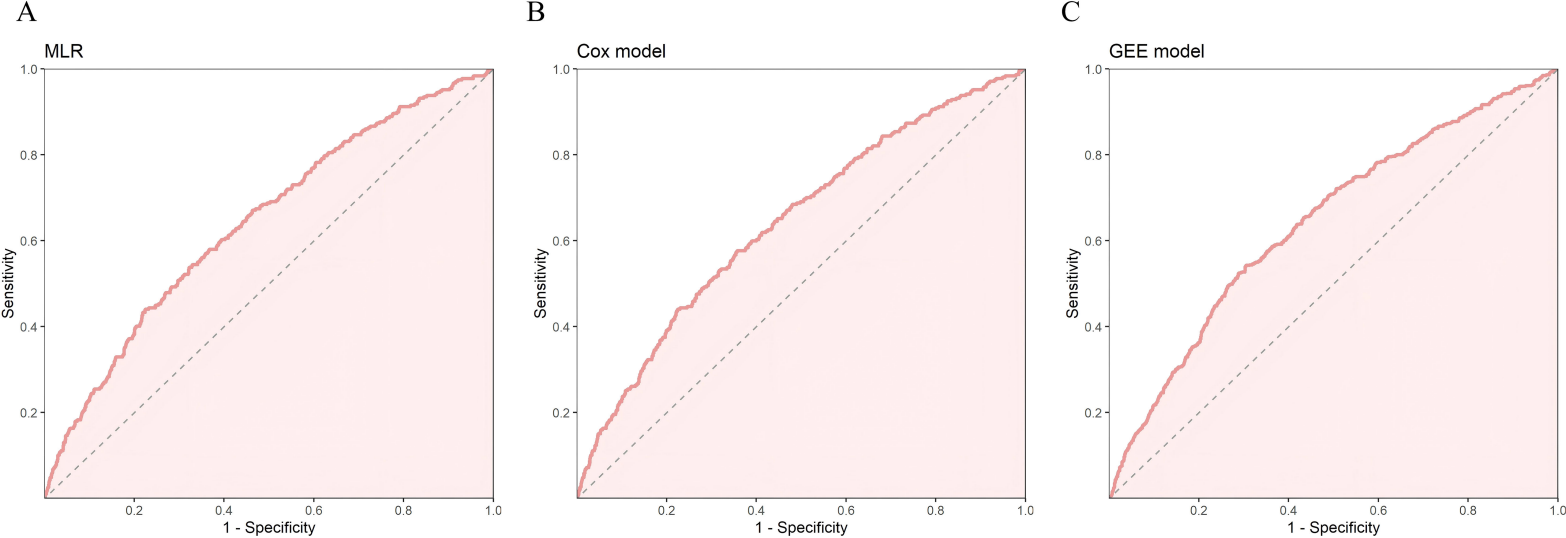
In the training set, **(A)** ROC curve of MLR, **(B)** ROC curve of Cox risk model, **(C)** ROC curve of Generalized Estimating Equations (GEE).

We evaluated the performance of the three models above using ROC curves. All three models had AUC values close to 0.64. The 95% confidence intervals of AUC for the MLR and Cox models ranged from 0.610 to 0.674, while the GEE model ranged from 0.623 to 0.662. In terms of the Youden index, the GEE model performed the best, reaching 0.237. For sensitivity, the Cox model performed the best (0.577), while for specificity, the MLR model had the highest performance (0.776). The GEE model performed relatively well in balancing sensitivity and specificity, while the MLR model had the best specificity, and the Cox model had the best sensitivity. We selected the five most significant factors from the three models, which were NLR, FPG, Cr, BMI, and Exercise Habits (**Table 4**) (**Figures 2A-C**).

### 3.6 Nomogram construction and evaluation

**Figure 3:**
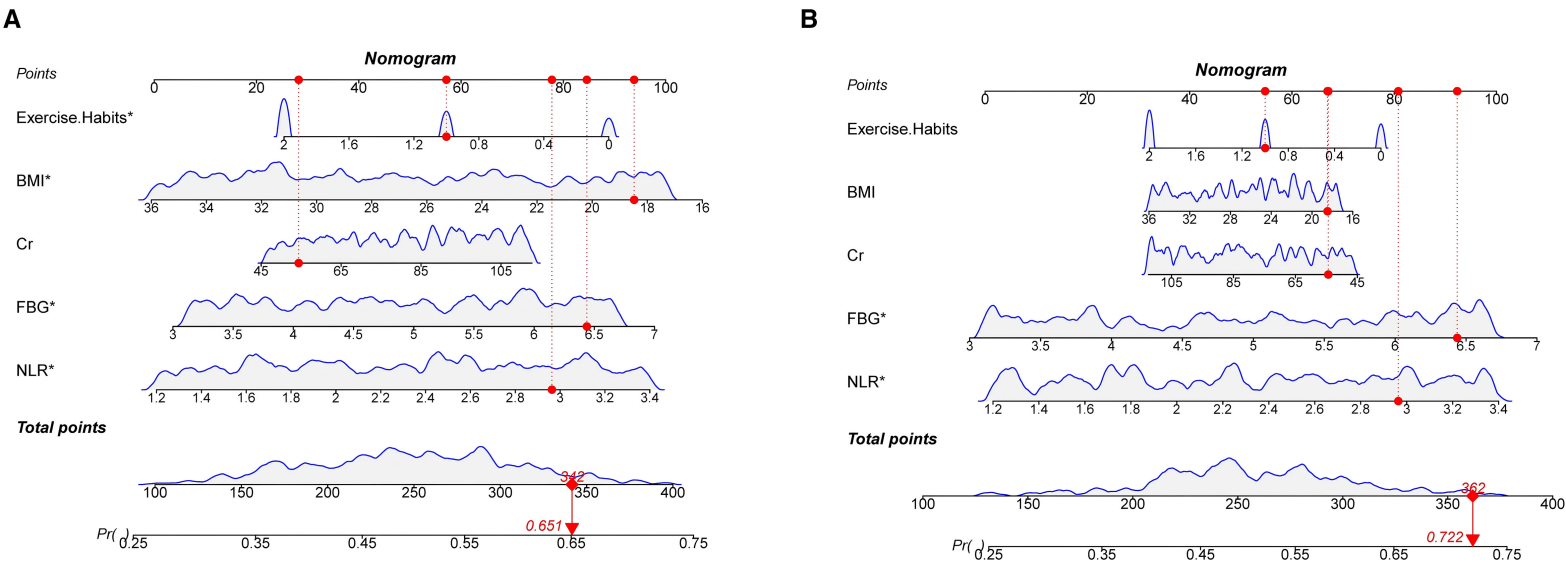
**(A)** Nomogram of the training set and **(B)** Nomogram of the test set.

**Figure 4:**
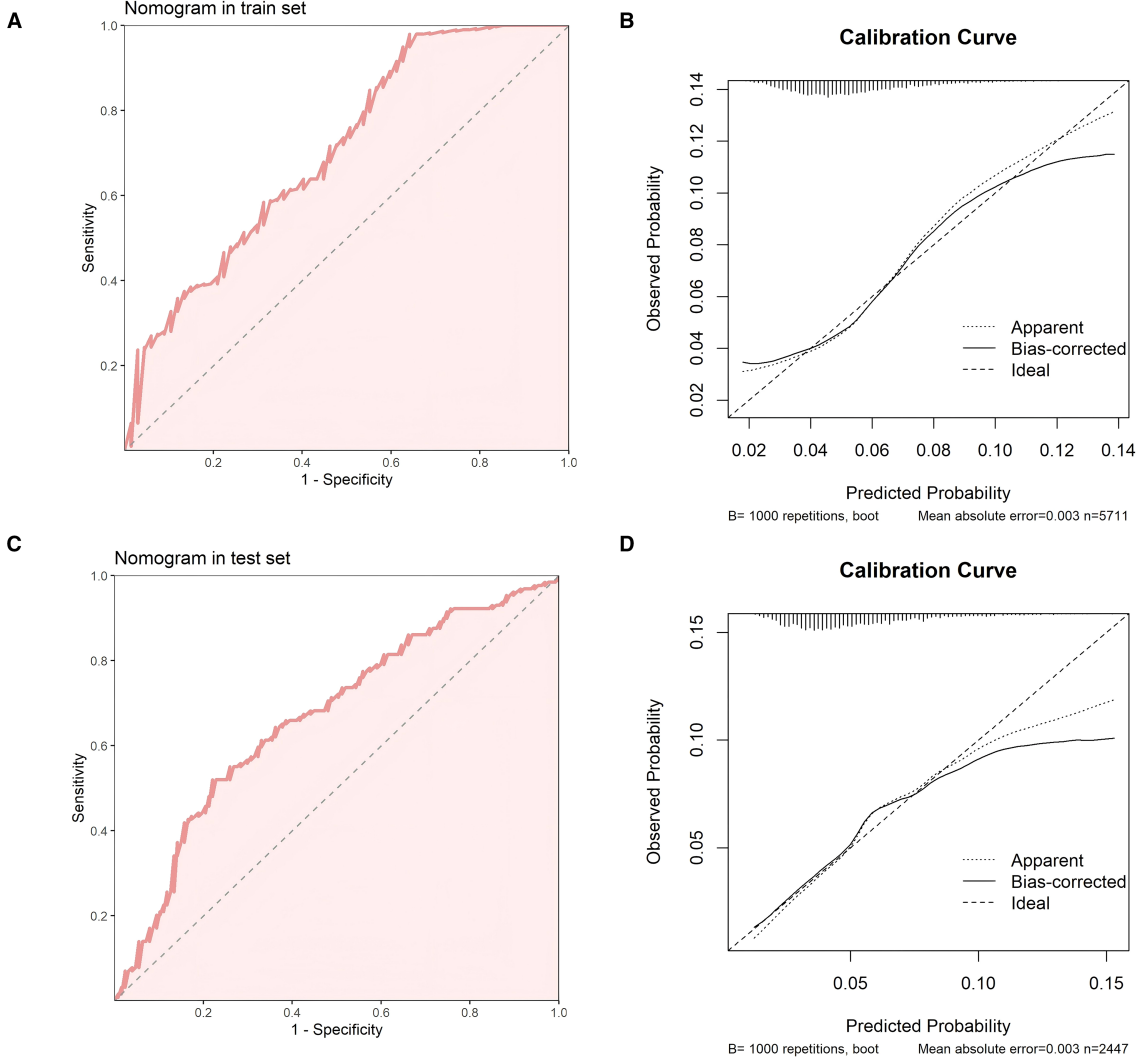
**(A)** ROC curve of the nomogram in the training set **(B)** Calibration curve of the nomogram in the training set. **(C)** ROC curve of the nomogram in the test set **(D)** Calibration curve of the nomogram in the test set.

We constructed a nomogram in the training set based on the five most significant factors and validated it in the test set. Using these indicators, the probability of a patient developing diabetes within the next 5 years can be predicted. For example, in the training set, the patient’s total score is 342, and the probability of developing diabetes is 65.1% (**Figures 3A, B**). The AUC of the nomogram model in the training set is 0.705, and the calibration curve is close to the ideal 45-degree diagonal, demonstrating a robust predictive ability. Similarly, the AUC and calibration curve of the nomogram in the test set also show good predictive ability (**Table 4**) (**Figures 4A-D**).

**Table 4:** ROC curve parameters.

	AUC	AUC_CI_Lower	AUC_CI_Upper	Best_Threshold	youden	Sensitivity	Specificity
MLR	0.642	0.610	0.674	0.073	0.215	0.440	0.776
Cox	0.642	0.610	0.674	0.461	0.220	0.577	0.643
GEE	0.643	0.623	0.662	0.039	0.237	0.542	0.695
Train Set	0.686	0.653	0.720	0.062	0.182	0.563	0.719
Test Set	0.666	0.600	0.733	0.048	0.299	0.519	0.780
Train Set	0.705	0.633	0.778	0.251	0.338	0.980	0.358

## 4 Discussion

Prevalence analysis indicates that the incidence of newly diagnosed diabetes in 2020–2021 was higher than in the previous two years, which may be related to various social factors, most notably the impact of the COVID-19 pandemic. The peak of the pandemic occurred during 2020 and 2021, bringing multiple pressures—social, economic, and psychological—to people, thereby influencing their lifestyles. During this period, many people chose to stay at home, reducing daily activities and leading to unhealthy routines. Some individuals also developed mental health issues such as anxiety and depression, which could cause metabolic disorders and indirectly increase the risk of diabetes.

NLR, as a simple indicator of the body’s inflammatory state, primarily reflects immune inflammation, which plays a crucial role in the development of diabetes (23). Chronic low-grade inflammation is considered an important mechanism for the occurrence and progression of diabetes, possibly by weakening the insulin sensitivity of insulin target tissues (such as adipose tissue, muscle, and liver), leading to insulin resistance (IR) (24). In addition, inflammation activates several signaling pathways (such as NF-κB), inhibiting insulin signal transduction. Similarly, the increase in PLR reflects a systemic inflammatory state, with increased pro-inflammatory cytokines (such as IL-6 and TNF-α) interfering with insulin signaling pathways, promoting IR (25). The increase in platelet count in PLR suggests platelet activation, releasing pro-inflammatory mediators (such as CD40L, P-selectin) (26), exacerbating endothelial inflammation and oxidative stress, further promoting IR. The decrease in lymphocytes may reflect immune regulation abnormalities, and some studies have shown that the lack of regulatory T cells can induce IR (27). FPG, which is measured after at least 8 hours of fasting, is a key diagnostic criterion for diabetes, with levels greater than 7.0 mmol/L indicating the presence of diabetes. Cr is a waste product generated by muscle metabolism, often associated with kidney function impairment (28). Elevated Cr levels may indicate some degree of kidney dysfunction, which is often subtle and not easily detected. Since the kidneys play a critical role in insulin metabolism, kidney dysfunction means insulin is not fully metabolized, leading to elevated insulin levels and increased risk of metabolic disorders. High BMI, or obesity, is closely linked to the onset of diabetes (29). Extensive research has shown that abdominal obesity leads to the secretion of various hormones and cytokines, which induce chronic low-grade systemic inflammation, thus increasing the risk of insulin resistance (30). Adipose tissue significantly affects glucose metabolism; fat cells participate in the storage and metabolism of fatty acids, and the more fat cells there are, the higher the concentration of fatty acids in the blood. These fatty acids interfere with insulin action, leading to insulin resistance (31), which, if persistent, is likely to develop into diabetes. We also found that exercise habits are closely related to diabetes; the diabetes incidence rate is significantly higher in individuals who lack physical activity. This may be because exercise promotes the utilization and transport of blood glucose, and both aerobic exercise and strength training help muscle cells absorb glucose. Regular exercise can effectively reduce body fat and enhance insulin responsiveness, allowing cells to use insulin more efficiently to absorb glucose, thus lowering the risk of diabetes (32). Regular exercise also helps regulate hormone balances closely related to diabetes, such as leptin and adrenaline, further reducing the risk of diabetes.

The innovation of this study lies in the use of multiple multivariate analysis methods, including multivariate logistic regression, Cox proportional hazards model, and generalized estimating equations. The combination of these methods not only enhances the reliability and validity of the results but also provides scientific evidence for early prevention and intervention of diabetes. It allows for a multidimensional exploration of the pathogenesis of diabetes and offers a reference for disease prevention.

This study also has some limitations. Firstly, the data were only from Wuxi, China, which introduces regional bias, and thus the findings may not be widely generalizable to other regions or different age and ethnic groups. Secondly, as a retrospective study, there may be some selection bias in the data. Additionally, the study did not include all potential factors that could affect the incidence of diabetes. Finally, some variables, such as smoking, alcohol consumption, and physical activity habits, relied on self-reports from patients, which could be influenced by recall bias or social desirability bias. Although factors such as gender did not show significant effects in this study, future research could further explore their potential impact on the incidence of diabetes. Additionally, gender differences may vary across different regions or ethnic groups, and these factors should be considered in future studies.

The nomogram developed in this study is an effective tool for predicting the risk of future diabetes onset. This model can provide scientific evidence for public health decision-making, particularly in early intervention and personalized management. Clinically, based on this model, doctors can accurately identify high-risk populations in advance and implement early intervention measures, thereby delaying or preventing the onset of diabetes. In the future, the model can be further validated for its applicability and robustness in different regions, ethnic groups, and age cohorts. Furthermore, with technological advancements, the model can be optimized by integrating more clinical, genetic, and environmental factors to improve predictive accuracy. In-depth exploration of the mechanisms of new-onset diabetes, especially the interactions between different factors, will help identify early signals of diabetes more precisely. Combining big data and artificial intelligence technologies, future research could explore how to better apply such predictive tools in daily health management, thereby improving the overall level of public health management.

## 5 Conclusion

This study shows that the incidence of newly diagnosed diabetes was highest between 2020 and 2021 during the period from 2018 to 2023. Significant factors influencing the onset of diabetes include NLR, FPG, Cr, BMI, and exercise habits. The nomogram constructed based on these factors demonstrates good predictive ability.

## Data Availability

The raw data supporting the conclusions of this article will be made available by the authors, without undue reservation.

## Supplementary material

Supplementary Figure 1 The research flowchart.
