# Supplementary figures and images for: Prevalence of diabetes and prediabetes among working-age adults and influencing factors of new-onset diabetes: a five-year cohort study (2018–2023)
Source: Front Endocrinol (Lausanne). 2025 Sep 17;16:1626925. doi: 10.3389/fendo.2025.1626925 (PMC12483861; doi:10.3389/fendo.2025.1626925)

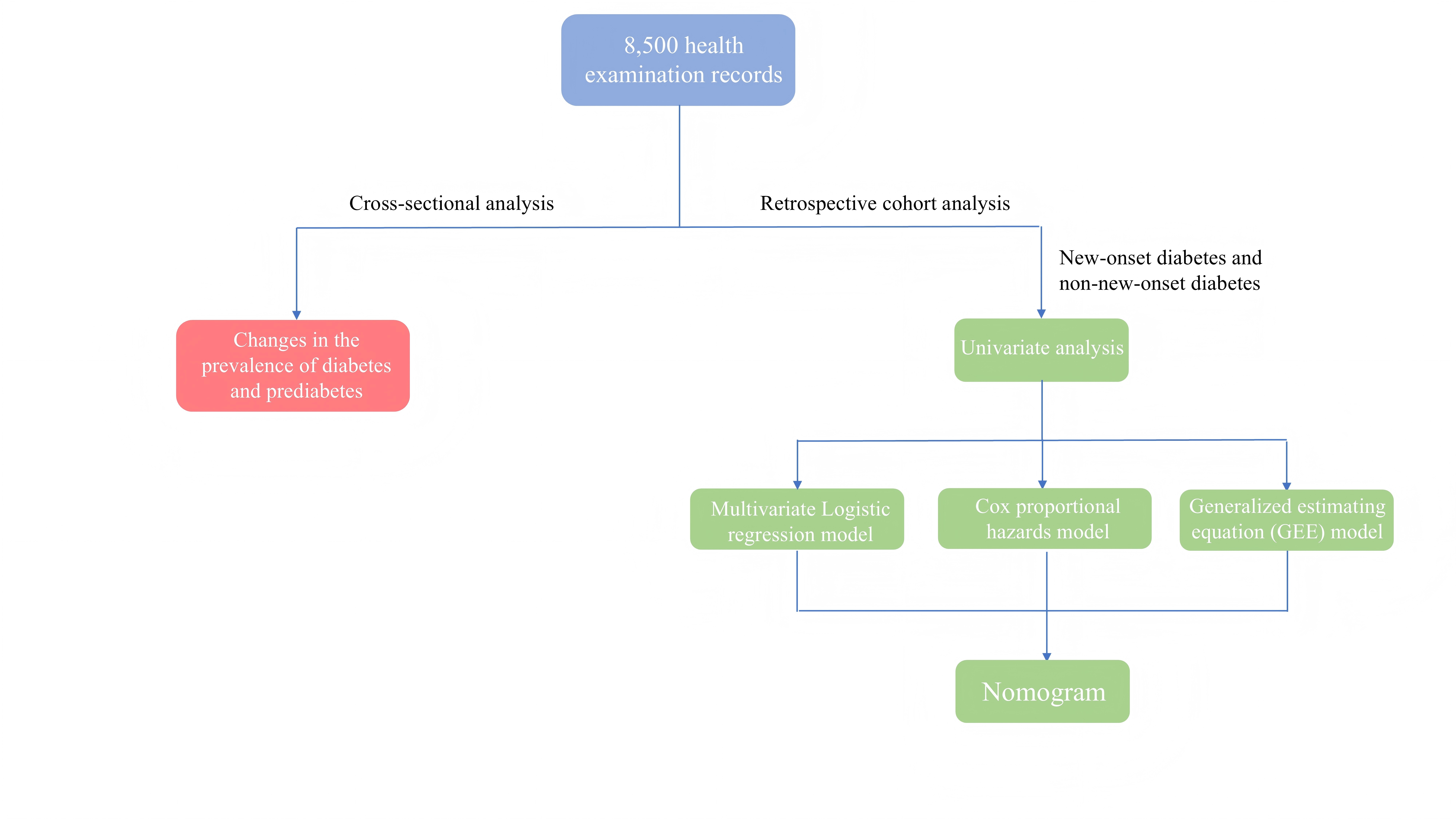

Supplement: Supplementary Figure 1 — The research flowchart. [file Image1.jpg]
