# Supplementary material for: Prevalence of diabetes and prediabetes among working-age adults and influencing factors of new-onset diabetes: a five-year cohort study (2018–2023)
Source: Front Endocrinol (Lausanne). 2025 Sep 17;16:1626925. doi: 10.3389/fendo.2025.1626925 (PMC12483861; doi:10.3389/fendo.2025.1626925)
Supplement: Supplementary file 2 [file Table1.docx]

| **Supplement Table. 1 Variance Inflation Factors (VIF) of Independent Variables** | |
| --- | --- |
| **Variables** | **Variance Inflation Factor (VIF)** |
| BMI | 1.002 |
| FBG | 1.002 |
| HbA1c | 1.003 |
| TC | 1.002 |
| TG | 1.002 |
| FI | 1.001 |
| TBIL | 1.002 |
| Cr | 1.002 |
| eGFR | 1.001 |
| WBC | 1.002 |
| NLR | 1.005 |
| PLR | 1.002 |
| Residential Area | 1.003 |
| Exercise Habits | 1.003 |
